# Variant Ataxia–Telangiectasia Presenting as Tremor–Dystonia Syndrome in a Bulgarian Religious Minority

**DOI:** 10.3390/genes16060641

**Published:** 2025-05-27

**Authors:** Teodora Chamova, Tihomir Todorov, Paulius Palaima, Petya Yankova, Iliyana Pacheva, Ivan Ivanov, Bilyana Georgieva, Sylvia Cherninkova, Alexey Savov, Dora Zlatareva, Elisaveta Naumova, Albena Todorova, Albena Jordanova, Ivailo Tournev

**Affiliations:** 1 Department of Neurology, University Hospital “Alexandrovska”, Medical University, 1431 Sofia, Bulgaria; s_cherninkova@abv.bg (S.C.); itournev@emhpf.org (I.T.); 2 Genetic Medico-Diagnostic Laboratory “Genica”, 1431 Sofia, Bulgaria; tisho.todorov@abv.bg (T.T.); todorova_albena@abv.bg (A.T.); 3 Molecular Neurogenomics Group, VIB Center for Molecular Neurology, VIB, 2610 Antwerp, Belgium; ppalaima@gmail.com (P.P.); albena.jordanova@uantwerpen.be (A.J.); 4 Molecular Neurogenomics Group, Department of Biomedical Sciences, University of Antwerp, 2610 Antwerp, Belgium; 5 Department of Clinical Immunology, University Hospital “Alexandrovska”, Medical University, 1431 Sofia, Bulgaria; petya_yankova@yahoo.com (P.Y.); naumovaej@gmail.com (E.N.); 6 Department of Pediatrics, University Hospital “St George”, 4000 Plovdiv, Bulgaria; inapatcheva@gmail.com (I.P.); ivanovist@gmail.com (I.I.); 7 Department of Pediatrics, Medical University of Plovdiv, 4000 Plovdiv, Bulgaria; 8 Research Institute at Medical University of Plovdiv, 4000 Plovdiv, Bulgaria; 9 Department of Medical Chemistry and Biochemistry, Medical University, 1431 Sofia, Bulgaria; bgeorgieva@medfac.mu-sofia.bg; 10 National Genetic Laboratory, Department of Obstetrics and Gynecology, Faculty of Medicine, Medical University, 1431 Sofia, Bulgaria; asavov@medfac.acad.bg; 11 Department of Diagnostic Imaging, University Hospital “Alexandrovska”, Medical University, 1431 Sofia, Bulgaria; dorazlat@yahoo.com; 12 Department of Cognitive Science and Psychology, New Bulgarian University, 1618 Sofia, Bulgaria

**Keywords:** tremor–dystonia, *ATM* gene, Bulgarian Muslims, WES

## Abstract

**Background:** Ataxia-telangiectasia (A-T) is a rare autosomal recessive disorder due to mutations in the *ATM* gene. Given the residual kinase activity and the type of *ATM* mutation, its clinical spectrum varies from a severe classic phenotype to a variant atypical form. **Material and methods:** This study included 28 patients belonging to four big Bulgarian Muslim pedigrees with tremor and dystonia. Whole-exome sequencing was performed in seven affected individuals from two unrelated pedigrees, followed by Sanger sequencing of the coding sequences and exon–intron borders of the *ATM* gene. **Results:** Twenty-four of the affected individuals were homozygous for c.8147T>C (p.Val2716Ala) in *ATM*, while four of the affected individuals were compound heterozygous. The targeted Sanger sequencing along the *ATM* gene revealed as a second mutation in three of the patients the splice-site variant c.4909+1G>A and in one patient a synonymous pathogenic variant with a splicing effect, c.3576G>A, p.Lys1192. The age at onset in our group varied between 14 days and 40 years. The main symptoms were dystonia and tremor, more prominent in the upper limbs and the neck, and dystonic dysarthria and dysphagia. The clinical course was very slowly progressive. Brain imaging was normal in the majority of the patients. **Conclusion:** Clinical features due to mutations in the *ATM* gene can be very broad. The disease may appear as dystonia, especially of early onset, without frank cerebellar involvement and also normal cerebral imaging. A-T should be considered in all patients with unexplained, even mild movement disorders and elevated α fetoprotein.

## 1. Introduction

Ataxia–telangiectasia is a rare autosomal recessive disorder caused by mutations in the *ATM* gene, located on chromosome 11q22.3 (MIM 208900) [1]. This gene is known to harbor 66 exons and encodes a PI3K family kinase with 3050 amino acid residues. Currently, more than 1400 unique variants have been identified in this gene [2]. The ataxia–telangiectasia-mutated (ATM) protein is a serine-threonine protein kinase, which phosphorylates more than 700 substrates and is a key player in the cellular response to double-stranded DNA damage [3]. Due to the residual kinase activity and the nature of the *ATM* mutation, the clinical presentation of AT can vary widely—from the classic form characterized by severe, early-onset, and rapidly progressive neurodegeneration to an atypical variant with milder neurological symptoms and a reduced risk of systemic complications. Patients with classic ataxia–telangiectasia present with a severe phenotype due to absent ATM kinase activity [4], either attributed to two null mutations or mutations that result in protein without ATM kinase activity. Affected individuals have severe multisystem involvement, including cerebellar ataxia, tremor, dystonia, oculomotor dyspraxia, polyneuropathy, immunological defects, respiratory problems, oculocutaneous telangiectasia, and an increased risk of malignancy [5,6,7]. Most patients with classic ataxia–telangiectasia lose ambulation during adolescence and die before the age of 30 due to malignancy or respiratory failure.

The term ‘‘variant A-T’’ is used to describe patients with milder phenotypes, associated with missense or splice-site mutations that produce ATM proteins with some residual kinase activity [8]. The variant A-T patients have a milder late-onset atypical phenotype characterized by a predominance of tremor, dystonia, ataxia with later onset, and slower neurologic progression. Although respiratory diseases and immunodeficiency are less severe in variant A-T, the risk of malignancy remains high [4,6,9].

Here, we report the clinical features of a cohort of 28 patients with variant ataxia–telangiectasia caused by the missense c.8147T>C, p.Val2716Ala and the splice-site c.4909+1G>A and c.3576G>A, p.Lys1192 variants and who belong to a religious minority from the southwest of Bulgaria.

## 2. Materials and Methods

This study encompassed 28 patients (12 male and 16 female) from four unrelated pedigrees belonging to a religious minority in Bulgaria. The families were identified through field studies and records from the Department of Neurology at the Medical University of Sofia. All participants provided written informed consent. This study adheres to the ethical guidelines of the participating institutions. It was approved by the Ethics Committee of Sofia Medical University, 4873, approved on 9 July 2018.

All the affected patients underwent clinical neurological assessment, including the Scale for Assessment and Rating of Ataxia (SARA) and Unified Dystonia Rating Scale (UDRS) [10,11]. According to the predominant neurological symptoms, the patients were divided into three neurological phenotypic groups: Group 1, cerebellar ataxia with minimal or no extrapyramidal involvement; Group 2, cerebellar ataxia combined with additional extrapyramidal features; and Group 3, extrapyramidal signs without notable ataxia [12].

Each patient’s medical history, including any instances of diabetes and malignancy, was thoroughly documented.

Height and weight were collected from all of the affected individuals and compared to the normative values of the Bulgarian population.

Neuro-ophthalmological assessment for conjunctival telangiectasia and eye movement abnormalities was performed in all of the affected patients. Nerve conduction studies were conducted in 16 of the affected individuals. 

MRI brain scans were conducted by a 3 Tesla MRI unit (Siemens Verio) in 14 patients from this cohort. Brain CT scans were conducted in 3 affected patients. Alpha fetoprotein in blood (AFP) was tested in 14 patients. 

### 2.1. Immunological Assessment

History of infections was assessed in all patients. Immunological examination of four patients was performed, including the immunophenotyping of lymphocytes (T lymphocytes and their subpopulations: Ts, Th, Th17, and Treg; B and NK cells), T cell function during stimulation with PHA and anti-CD3/CD28 dynabeads, determination of immunoglobulins (IgG and their subclasses,: IgA, IgM, and IgE), complement fractions (C3 and C4), antinuclear antibodies (ANA), and post-vaccinal immune response against tetanus and diphtheria anatoxin. Indirect immunofluorescence (IIF) with HEp-2 cells was used as a screening technique for the detection of antinuclear autoantibodies. Flow cytometry analyses were used to detect lymphocyte populations and functional markers on T cells [13].

### 2.2. Whole-Exome Sequencing and Homozygosity Mapping

**Figure 1:**
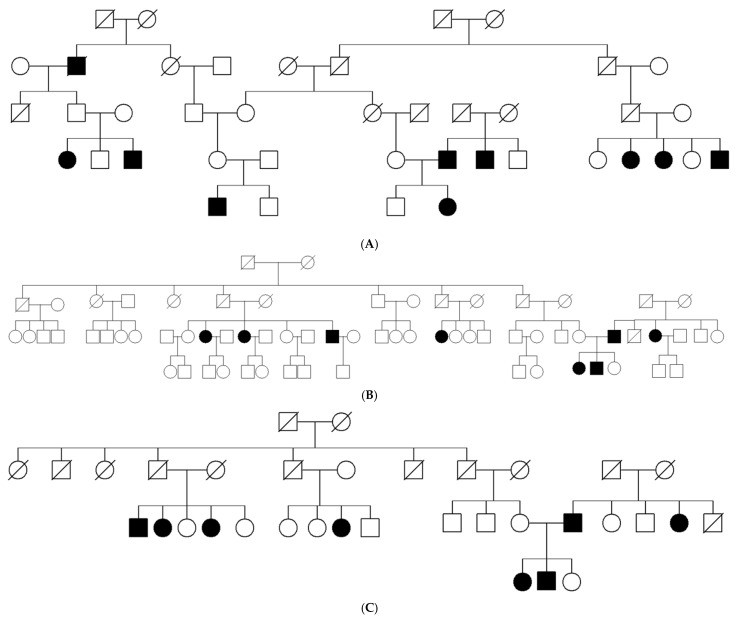
(**A**–**C**) Pedigrees of the affected Bulgarian Muslims.

Genomic DNA was isolated from peripheral blood mononuclear cells following standard procedures. Whole-exome sequencing (WES) was performed in two affected individuals and their parents from Family 1 from the town of Sarnitza and two affected siblings and their mother from Family 2 from the town of Dospat (Figure 1). Exome capture and enrichment was conducted using a SureSelect Human All Exon v5.0 kit (Agilent Technologies, Santa Clara, CA, USA), followed by paired-end sequencing on the HiSeq4000 platform (Illumina, San Diego, CA, USA). Primary analyses were conducted using the GenomeComb pipeline [14], which employed the Burrows–Wheeler algorithm [15] for read alignment to the GRCh37/HG19 reference human genome and genomic analysis toolkit (GATK) (version 3.3.0)22 for variant calling. Homozygosity mapping based on WES data was performed as described in [16] using the HOMWES tool from the GenomeComb package (version 0.11.0).20. Variant filtering and prioritization were performed using GenomeComb20 based on a recessive model of inheritance and the following criteria: variants within homozygous regions, non-synonymous or splice variants with a minor allele frequency (MAF) below 5%, and no homozygotes in the control population database gnomAD v2.1.1. [17]. Prioritized variants were evaluated further using Alamut Visual Plus (Sophia Genetics, Rolle, Switzerland).

### 2.3. Variant Segregation and Cohort Carrier Screening

The resulting variants were validated and segregated among the available family members using Sanger sequencing, as previously outlined.

Affected family members who turned out to be heterozygous for the variant c.8147T>C (p.Val2716Ala) were subjected to direct Sanger sequencing of the coding sequences and exon–intron borders of the *ATM* gene. The targeted screening along the *ATM* gene revealed two additional pathogenic variants, c.4909+1G>A and c.3576G>A, p.Lys1192, both of them affecting the normal splicing.

### 2.4. Screening in the Towns of Dospat and Surnica

Due to the fact that all patients belonged to a religious minority of Bulgarian Muslims originating from the Dospat and Surnica towns in the Rhodope Mountains, a selective screening targeting the most common mutation missense mutation c.8147T>C (p.Val2716Ala) was performed on newborns from each of both the presumable endemic regions. Altogether, 200 samples (100 for each region) were selected from a Guthrie card dry blood spot collection of national newborn screening programs (kindly provided by the National Genetic Laboratory, Sofia, Bulgaria). The study was approved by the Local Ethical Committee. 

The screening for the targeted mutation was performed by PCR amplification followed by Sanger sequencing by a BigDye^®^Terminator cycle sequencing kit v.3.1 (Applied Biosystems, Foster City, CA, USA) [18]. Primers were designed to specifically amplify the targeted exon 57 of the *ATM* gene (RefSeq NM_000051). The electrophoretic separation was performed on an ABI3130 Genetic Analyzer (Applied Biosystems, Foster City, CA, USA). The sequencing profiles were interpreted by the software Sequencing Analysis v5.1.1.

### 2.5. ATM Protein Expression

Total protein extracts were isolated from lymphoblast cells, separated on a custom-made 7.5% SDS-PAGE and transferred to blotting membranes as described in [19]. Membranes were incubated with the following primary antibodies: anti-ATM (Abcam, Cambridge, UK, 2C1 (1A1), 1:2000) and anti-actin (Merck, Darmstadt, Germany, A5441, 1:5000). Anti-mouse IgG1 (Southern Biotech, Birmingham, AL, USA, 1070-05, 1090-05, 1:10,000) was used as a secondary antibody. The blots were developed with Pierce ECL Plus substrate (Thermo Fisher Scientific, Waltham, MS, USA) and imaged using the Amersham Imager 600 (GE Healthcare, Chicago, IL, USA).

## 3. Results

**Figure 2:**
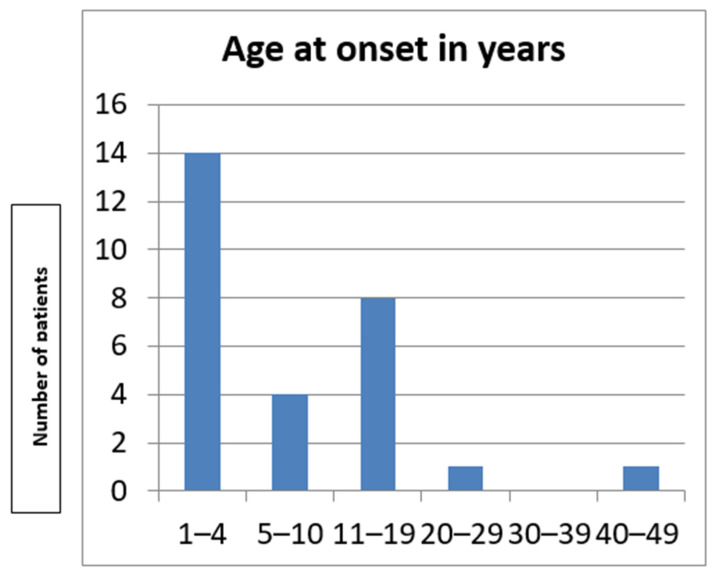
Age at onset in the Bulgarian cohort.

The study included 28 individuals (12 male and 16 female) from four unrelated pedigrees (Figure 1) belonging to a religious minority of Bulgarian Muslims inhabiting the region of the Dospat and Surnica towns in the Rhodope Mountains. The mean age was 8.3 years ± 9.3 years, varying between 14 days and 40 years (Figure 2).

**Figure 3:**
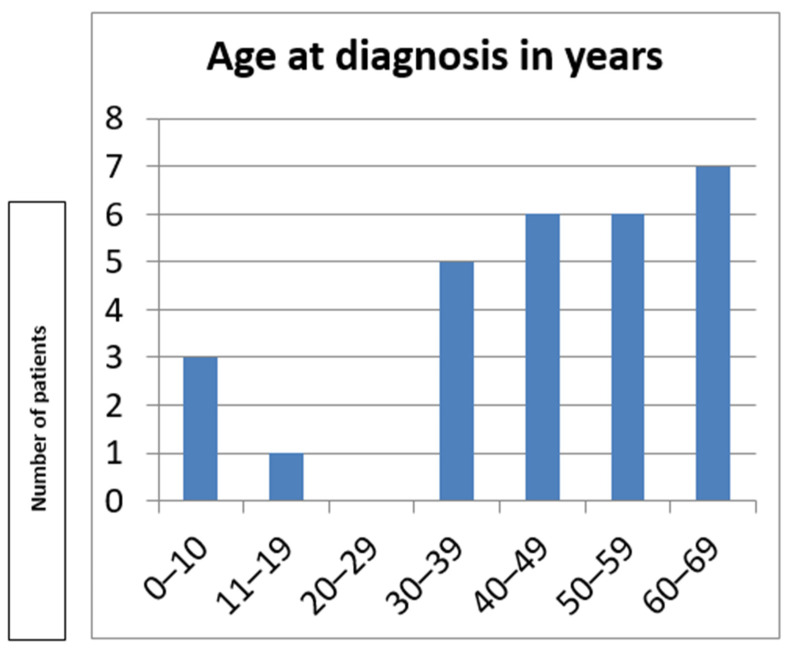
Age at diagnosis in the Bulgarian cohort.

Mean age at diagnosis was 44.75 ± 18.03 years, ranging from 8 to 69 years (Figure 3). The diagnostic delay ranged between 5 and 58 years after the initial symptoms. None of the patients were wheelchair-bound at the time of the last assessment. No growth retardation was present in our cohort.

**Figure 4:**
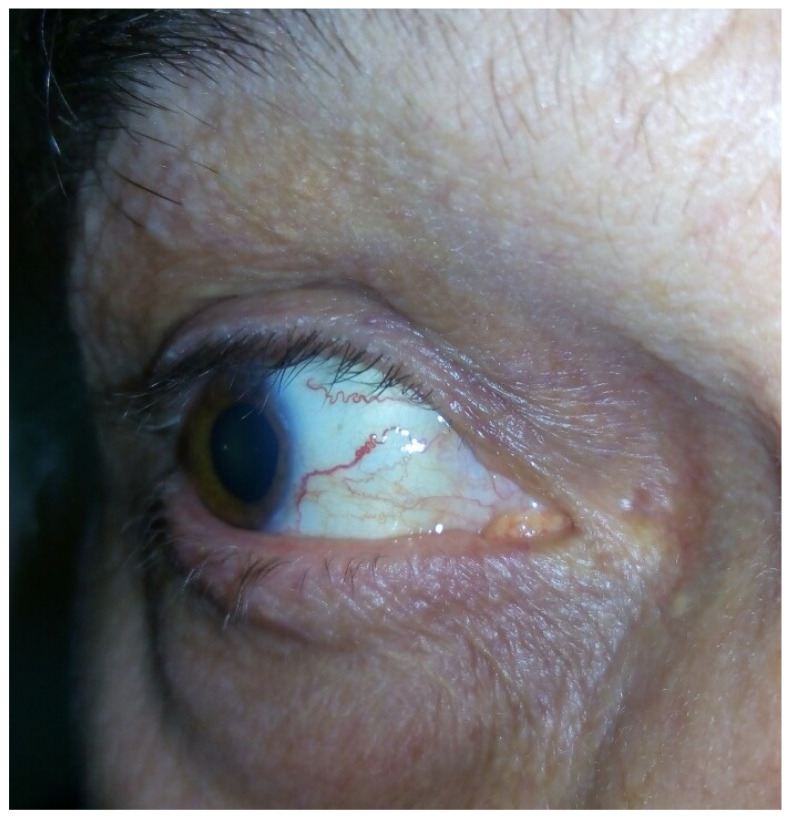
Conjunctival telangiectasia of patient 9, aged 63 years.

The main clinical features present in all of the affected individuals (Table 1) encompassed dystonia, resting, and postural tremor, predominantly affecting the head and the upper limbs (Supplementary Material). Dystonic dysarthria and dysphagia were observed in the more advanced stages. Chorea was observed in 10/28 of the patients, while myoclonus was present in 5/28. The movement disorders were slowly progressing over time. Mild ataxia of stance and gait was present only in 5/28 of the patients. All the affected individuals were ambulant at the time of their last assessment. In 4/28 patients, dilated conjunctival vessels were found (Figure 4).

Axonal polyneuropathy with decreased amplitudes of the peroneal and sural nerves was present in a small proportion of the patients: 4/17.

All the patients were classified in Group 3: extrapyramidal signs without notable ataxia and/or peripheral neuropathy. None of them were wheelchair-bound at the time of the last follow-up.

Brain MRI revealed cerebellar atrophy in only 1/17 of those tested (patient 28), who was compound heterozygous for p.V2716A and p.Lys119 in the *ATM* gene. Basal ganglia seemed unaffected as well.

Alpha fetoprotein was significantly increased in all of the 14 tested individuals, more than four times over the upper limit. None of the patients had a history of malignancy.

None of the patients reported severe infections during childhood. Although T cells were significantly diminished compared to standard values in three patients, the T cell subpopulations had a normal ratio. Only one patient (patient 28) showed severely reduced CD8+ T lymphocyte values. B cell lymphopenia and lower lymphocyte counts of regulatory T cells and elevated Th17 cell values were reported in all four patients. The immunological tests revealed normal expression of CD69+ total T lymphocytes with stimulation with PHA and anti-CD3/CD28 dynabeads and normal immunoglobulin and complement values. Non-protective post-vaccinal immune response against tetanus and diphtheria anatoxin and the titer of ANA was between 1/320 and 1/640. 

**Figure 5:**
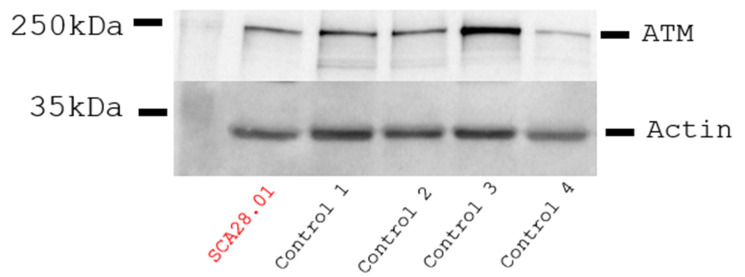
Immunoblotting of total protein lysates from EBV-transformed lymphoblasts derived from patient 11 (SCA28.01) and four male age-matched control individuals. No reduction in the ATM levels was detected in the patient compared to controls. Actin expression is used for band intensity normalization.

Initial whole-exome sequencing followed by homozygosity mapping was conducted in seven patients and their available parents, belonging to three multigenerational families. The HOMWES analysis distinguished six regions of homozygosity shared between all of the affected individuals, with the largest one (9.2 Mb) located on chromosome 11 (chr11:102098354-111325051). Variant filtering and prioritization within those regions revealed a homozygous missense variant NM_000051.4(*ATM*):c.8147T>C (p.Val2716Ala) in exon 57 of the *ATM* gene. The variant was confirmed by Sanger sequencing, and the segregation analysis of available relatives demonstrated that it co-segregated with the disease. This variant was previously associated with variant A-T (ref). The immunoblotting analysis of total protein lysates isolated from lymphoblastoid cells available from patient 5 (SCA28.01) revealed that the AT gene was expressed at comparable levels to the gender- and age-matched control individuals (Figure 5). 

Twenty-four of the affected patients were homozygous for c.8147T>C (p.Val2716Ala) in *ATM*, while four of the affected were compound heterozygous. The targeted Sanger sequencing along the *ATM* gene revealed as a second mutation in three of them the splice-site variant c.4909+1G>A and in one patient a synonymous pathogenic variant with a splicing effect, c.3576G>A, p.Lys1192.

Since there were cases in two consecutive generations (Figure 1) without a history of endogamy, the hypothesis of a high carrier rate of c.8147T>C (p.Val2716Ala) in *ATM* gene was tested. Altogether, 200 newborns originating from Dospat and Surnica were tested for the targeted mutation, c.8147T>C (p.Val2716Ala). The results showed three heterozygous carriers, which represents a carrier frequency in this subpopulation of about 1.5% (3/200). The mutant allele frequency was calculated on the base of 400 alleles screened, which revealed 0.75% (3/400). 

## 4. Discussion

We present a large cohort of patients with a milder clinical phenotype, known as the A-T variant, belonging to a religious minority of Bulgarian Muslims. All individuals included in this study had neurological involvement, while systemic features were either mild or absent, which is in line with previous reports [12,20,21].

Despite genetic homogeneity in the *ATM* gene variants within our group, the age of onset varied widely, ranging from 14 days to 40 years. Such variation was observed in a group of Canadian Mennonites [20]. Consistent with other reports, tremor and dystonia, especially with facial, cervical, and brachial distribution, were invariably present in all the patients from the Bulgarian cohort [12,20], followed by chorea and myoclonus, observed in less than 50% of the affected patients in our group. They may occur without frank ataxia and may be misdiagnosed in adults with primary-appearing dystonia or essential tremor [12,20]. Mild ataxia was present in only 5/28 of the affected patients and did not progress significantly over time compared to the other movement disorders [21]. Moeini Shad et al. reported that patients with predominantly dystonic symptoms typically exhibit later disease onset and a milder clinical course—a pattern also observed in the Bulgarian cohort. However, it remains uncertain whether dystonia is associated with a specific genotypic abnormality [21].

In imaging of variant atypical AT patients, cerebellar morphology is often normal [22], as in our group only one patient had cerebellar atrophy on MRI.

It was reported that individuals with a missense mutation, which causes the production of a mutant ATM protein with retained kinase activity, are approximately five times more likely to have a malignancy than individuals in the other genetic groups due to a possible gain-of-function mechanism [12,20]. On the contrary, in our cohort, no malignancies have been reported up to now, but they are being thoroughly followed up.

AFP was elevated in all of the tested individuals from our cohort and may be considered as a useful screening tool with high sensitivity for A-T.

Immunodeficiency, though highly variable, is a hallmark of 60%–80% of patients with classic AT [3]; however, it is infrequently seen in variant forms of the disease [6]. We performed FACS analyses of T, B, and NK T cells from peripheral blood of four patients with the A-T variant to examine the expression of activation and functional markers. We observed a decrease in naïve helper (CD4+CD45RA+) cells, CD8+ T cells, and B cells and increased levels of Th17 cells, while the amount of CD3-CD56+ NK cells and CD3+CD56+ NKT-like cells was within the reference values corresponding to age. Despite the small number of patients studied, the results obtained are comparable to the results obtained by Graafen et al. [23]. In reviewing the available literature on elevated ANA values and their association with paraneoplastic syndrome in patients with the A-T variant, we found several findings that may confirm our results.

The most common mutation among this group, observed in a homozygous state in 24 affected patients, is p.Val2716Ala. The majority of Bulgarians are traditionally Christian, with only about 2% of the ethnic Bulgarian population belonging to the Muslim minority. This group, primarily concentrated in the Rhodope Mountains in the southwestern region of the country, was estimated at approximately 29,000 people according to the latest population census. This is the second genetic disorder after LGMD2G/R7 with a high frequency among this group [24,25]. In this region, the carrier rate of p.Val2716Ala was found to be quite high at 1.5% (3/200), probably due to cumulative effects of historical endogamy in this closed religious minority [24]. Our results indicate an expected high recurrence risk of variant ataxia–telangiectasia in these particular Bulgarian regions, which presumes the disease might be much more frequent than expected for the general population. These findings are important for the Muslim communities in the affected regions of Bulgaria. They could benefit from early genetic testing and appropriate genetic counseling during family planning.

## 5. Conclusions

Although there is no universally accepted definition of the variant AT phenotype, our findings align with previous reports indicating that variant A-T is characterized by tremor and dystonia, with or without overt ataxia. Because these cases are often underdiagnosed or misdiagnosed, early diagnosis through newborn screening and targeted testing in high-risk populations in Bulgaria is essential. This is particularly important given the associated risks of malignancies, as well as for facilitating genetic counseling, carrier detection within affected families, and prenatal diagnosis. 

## Figures and Tables

**Table 1:** Clinical features of the Bulgarian patients with variant A-T.

Patient Number	Genotype	Sex	Date of Birth	Age at Onset	Dystonia				Tremor			Dystonic Dysarthria	Dystonic Dysphagia	Ataxia	Pyramidal Involvement	Telangiectasia	Neoplasms	Immune Deficiency	Alpha Fetoprotein	Brain MRI
					Face	Neck and Trunk	UL	LL	Head	UL	LL									
1	p.V2716A/p.V2716A	F	27.10.1973	17 y	-	-	-	R	+	+	-	-	-	-	+	-	-	-	ND	Normal
2	p.V2716A/p.V2716A	M	02.10.1976	16 y	-	-	-	R	+	+	-	+	-	-	-	-	-	-	43.01	Normal
3	p.V2716A/p.V2716A	M	17.06.1948	20 y	-	-	R > L	+	-	-	-	+	-	-	-	-	-	-	ND	ND
4	p.V2716A/p.V2716A	F	16.08.1955	7 y	+	+	R > L	+	-	R	-	-	-	-	+	-	-	-	56.8	Cerebral and cerebellar atrophy
5	p.V2716A/p.V2716A	F	19.06.1963	2 y	+	+	R > L	L > R	+	+	-	+	+	-	+	-	-	-	39.3	ND
6	p.V2716A/p.V2716A	M	25.10.1970	1 y	+	+	+	-	+	+	-	+	-	-	-	-	-	-	58.8	ND
7	p.V2716A/p.V2716A	F	04.06.1971	14 days	+	+	R > L	+	+	+	-	+	+	Ny	-	-	-	-	48.16	Normal
8	p.V2716A/p.V2716A	M	19.04.2005	1 y	-	+	+	-	-	-	-	+	-	-	-	-	-	-	ND	Normal
9	p.V2716A/p.V2716A	M	26.09.1957	18 y	-	+	R	-	-	-	-	+	-	-	-	+	-	-	8.5	Normal
10	p.V2716A/p.V2716A	M	03.01.1956	18 y	-	-	-	-	+	R > L	-	+	-	-	-	-	-	-	ND	Normal
11	p.V2716A/p.V2716A	F	05.06.1986	18 y	-	+	-	-	+	R > L	-	-	-	-	+	-	-	-	ND	Normal
12	p.V2716A/p.V2716A	M	13.08.1975	1.3 y	-	+	R > L	L > R	-	-	-	+	-	-	-	-	-	-	ND	Normal
13	p.V2716A/p.V2716A	F	12.03.2012	1 y	-	+	-	-	-	-	-	-	-	-	-	-	-	-	ND	ND
14	p.V2716A/p.V2716A	M	14.04.2010	1 y	-	+	-	-	-	-	-	-	-	-	-	-	-	-	ND	ND
15	p.V2716A/c.4909+1G>A	M	06.06.1952	40 y	+	+	R > L	-	+	+	-	+	-	+	-	-	-	-	167	Normal
16	p.V2716A/p.V2716A	F	21.10.1985	13 y	+	+	L > R	-	+	+	+	-	-	-	-	-	-	-	60.8	Normal
17	p.V2716A/p.V2716A	F	03.01.1972	1 y	+	+	+	-	-	-	-	+	-	-	-	-	-	-	ND	ND
18	p.V2716A/p.V2716A	M	09.02.1966	1 y	+	+	-	-	+	+	-	-	-	-	-	+	-	-	ND	ND
19	p.V2716A/p.V2716A	F	03.02.1955	14 y	+	+	-	-	+	R > L	-	+	+	-	-	-	-	-	ND	ND
20	p.V2716A/c.4909+1G>A	F	08.01.1965	2 y	+	+	-	+	+	R > L	+	-	-	-	-	-	-	-	ND	ND
21	p.V2716A/c.4909+1G>A	M	24.01.1959	4 y	-	-	-	+	+	-	-	-	-	-	-	-	-	-	ND	ND
22	p.V2716A/p.V2716A	F	18.10.1984	1 y	-	-	-	-	+	L > R	-	+	-	-	-	-	-	-	112.4	Normal
23	p.V2716A/p.V2716A	F	31.01.2011	1 y	-	+	-	-	-	-	-	-	-	+/-	-	-	-	Frequent respiratory infections	18.9	Normal
24	p.V2716A/p.V2716A	F	04/01/1980	5 y	-	+	-	-	-	-	-	+	-	-	-	-	-	-	83.2	Normal
25	p.V2716A/p.V2716A	M	10.09.1965	17 y	+	+	+	+	+	+	+	+	+	-	-	-	-	-	32.9	Normal
26	p.V2716A/p.V2716A	F	05.03.1955	6 y	+	+	+	+	+	R > L	+	+	+	Intention tremor in the left hand	-	+	-	-	25.4	Normal
27	p.V2716A/p.V2716A	F	28.05.1967	6 y	-	-	-	-	+	+	-	+	-	-	-	-	+	-	ND	ND
28	p.V2716A/p.Lys119	F	12.05.29187	0.5 y	+	+	-	-	+	+	-	+	-	+	-	-	-	-	351.8	Cerebellar atrophy

Abreviations used: + present; - absent; y years; R right; L left; F female; M male;

## Data Availability

The original contributions presented in this study are included in the article. Further inquiries can be directed to the corresponding author.

## Supplementary Materials

The following supporting information can be downloaded at: https://www.mdpi.com/article/10.3390/genes16060641/s1, Video S1: Patient 7 with clinical features of dystonia, resting, and postural tremor, predominantly affecting the head and the upper limbs.

## Abbreviations

The following abbreviations are used in this manuscript:

ANA	Antinuclear antibodies
A-T	Ataxia-telangiectasia
MRI	Magnetic Resonance Imaging
SARA	Scale for Assessment and Rating of Ataxia
UDRS	Unified Dystonia Rating Scale
WES	Whole-exome sequencing

## References

[1] Gatti R.A., Berkel I., Boder E., Braedt G., Charmley P., Concannon P., Ersoy F., Foroud T., Jaspers N.G., Lange K. (1988). Localization of an ataxiatelangiectasia gene to chromosome 11q22-23. Nature.

[2] Shao L., Wang H., Xu J., Qi M., Yu Z., Zhang J. (2023). Ataxia-telangiectasia in China: A case report of a novel ATM variant and literature review. Front. Neurol..

[3] McKinnon P.J. (2012). ATM and the molecular pathogenesis of ataxia telangiectasia. Annu. Rev. Pathol..

[4] Verhagen M.M., Last J.I., Hogervorst F.B., Smeets D.F., Roeleveld N., Verheijen F., Catsman-Berrevoets C.E., Wulffraat N.M., Cobben J.M., Hiel J. (2012). Presence of ATM protein and residual kinase activity correlates with the phenotype in ataxia-telangiectasia: A genotype-phenotype study. Hum. Mutat..

[5] Sedgwick R.P., Boder E., Vinken P.J., Bruyn S.W. (1991). Ataxia telangiectasia. Handbook of Clinical Neurology, Hereditary Neuropathies and Spino-Cerebellar Atrophies.

[6] Verhagen M.M., Abdo W.F., Willemsen M.A., Hogervorst F.B., Smeets D.F., Hiel J.A., Brunt E.R., van Rijn M.A., Majoor Krakauer D., Oldenburg R.A. (2009). Clinical spectrum of ataxia-telangiectasia in adulthood. Neurology.

[7] Woods C.G., Taylor A.M. (1992). Ataxia telangiectasia in the British Isles: The clinical and laboratory features of 70 affected individuals. QJM Int. J. Med..

[8] Pearson T.S. (2016). More Than Ataxia: Hyperkinetic Movement Disorders in Childhood Autosomal Recessive Ataxia Syndromes. Tremor. Other Hyperkinet. Mov..

[9] van Os N.J., Jansen A.F., van Deuren M., Haraldsson A., van Driel N.T., Etzioni A., van der Flier M., Haaxma C.A., Morio T., Rawat A. (2017). Ataxia-Telangiectasia: Immunodeficiency and Survival. Clin. Immunol..

[10] Schmitz-Hübsch T., du Montcel S.T., Baliko L., Berciano J., Boesch S., Depondt C., Giunti P., Globas C., Infante J., Kang J.S. (2006). Scale for the assessment and rating of ataxia: Development of a new clinical scale. Neurology.

[11] Comella C.L., Leurgans S., Wuu J., Stebbins G.T., Chmura T. (2003). Dystonia Study Group. Rat. Scales Dystonia: A multicenter assessment. Mov. Disord..

[12] Schon K., van Os N.J.H., Oscroft N., Baxendale H., Scoffings D., Ray J., Suri M., Whitehouse W.P., Mehta P.R., Everett N. (2019). Genotype, extrapyramidal features, and severity of variant ataxia-telangiectasia. Ann. Neurol..

[13] Naumova E., Lesichkova S., Milenova V., Yankova P., Murdjeva M., Mihailova S. (2022). Primary immunodeficiencies in Bulgaria —Achievements and challenges of the PID National Expert Center. Front. Immunol..

[14] Reumers J., De Rijk P., Zhao H., Liekens A., Smeets D., Cleary J., Van Loo P., Van Den Bossche M., Catthoor K., Sabbe B. (2011). Optimized filtering reduces the error rate in detecting genomic variants by short-read sequencing. Nat. Biotechnol..

[15] Li H., Durbin R. (2009). Fast and accurate short read alignment with Burrows–Wheeler transform. Bioinformatics.

[16] Kancheva D., Atkinson D., De Rijk P., Zimon M., Chamova T., Mitev V., Yaramis A., Fabrizi G.M., Topaloglu H., Tournev I. (2016). Novel mutations in genes causing hereditary spastic paraplegia and Charcot-Marie-Tooth neuropathy identified by an optimized protocol for homozygosity mapping based on whole-exome sequencing. Genet. Med..

[17] Karczewski K.J., Francioli L.C., Tiao G., Cummings B.B., Alföldi J., Wang Q., Collins R.L., Laricchia K.M., Ganna A., Birnbaum D.P. (2020). The mutational constraint spectrum quantified from variation in 141,456 humans. Nature.

[18] Todorova A., Ashikov A., Beltcheva O., Tournev I., Kremensky I. (1999). C283Y Mutation and Other C-Terminal Nucleotide Changes in the G-Sarcoglycan Gene in the Bulgarian Gypsy Population. Hum. Mutat..

[19] Amor-Barris S., Høyer H., Brauteset L.V., De Vriendt E., Strand L., Jordanova A., Braathen G.J., Peeters K. (2021). HINT1 neuropathy in Norway: Clinical, genetic and functional profiling. Orphanet J. Rare Dis..

[20] Saunders-Pullman R., Raymond D., Stoessl A.J., Hobson D., Nakamura K., Nakamura T., Pullman S., Lefton D., Okun M.S., Uitti R. (2012). Variant ataxia-telangiectasia presenting as primary-appearing dystonia in Canadian Mennonites. Neurology.

[21] Moeini Shad T., Yazdani R., Amirifar P., Delavari S., Heidarzadeh Arani M., Mahdaviani S.A., Sadeghi-Shabestari M., Aghamohammadi A., Rezaei N., Abolhassani H. (2022). Atypical Ataxia Presentation in Variant Ataxia Telangiectasia: Iranian Case-Series and Review of the Literature. Front. Immunol..

[22] Micol R., Ben Slama L., Suarez F., Le Mignot L., Beauté J., Mahlaoui N., Dubois d’Enghien C., Laugé A., Hall J., Couturier J. (2011). CEREDIH Network Investigators. Morbidity and Mortality From Ataxia-Telangiectasia are Associated With ATM Genotype. J. Allergy Clin. Immunol..

[23] Graafen L., Heinze A., Albinger N., Salzmann-Manrique E., Ganß F., Hünecke S., Cappel C., Wölke S., Donath H., Trischler J. (2024). Immune profiling and functional analysis of NK and T cells in ataxia telangiectasia. Front. Immunol..

[24] Chamova T., Bichev S., Todorov T., Gospodinova M., Taneva A., Kastreva K., Zlatareva D., Krupev M., Hadjiivanov R., Guergueltcheva V. (2018). Limb Girdle Muscular Dystrophy 2G in a religious minority of Bulgarian Muslims homozygous for the c.75G>A, p.Trp25X mutation. Neuromuscul. Disord..

[25] Atkinson D., Chamova T., Candayan A., Kastreva K., Asenov O., Litvinenko I., Estrada-Cuzcano A., De Vriendt E., Kukushev G., Tournev I. (2024). Identification and Characterization of Novel Founder Mutations in NDRG1: Refining the Genetic Landscape of Charcot–Marie–Tooth Disease Type 4D in Bulgaria. Int. J. Mol. Sci..

